# Spatially Resolved Protein Binding Kinetics Analysis in Microfluidic Photonic Crystal Sensors

**DOI:** 10.3390/s23125637

**Published:** 2023-06-16

**Authors:** Stefanie Lehmann, Fabio Aldo Kraft, Martina Gerken

**Affiliations:** Integrated Systems and Photonics, Faculty of Engineering, Kiel University, 24118 Kiel, Germany; fkr@tf.uni-kiel.de (F.A.K.); mge@tf.uni-kiel.de (M.G.)

**Keywords:** photonic crystal biosensor, protein binding kinetics, spatially resolved spectral analysis, microfluidics, diffusion, label-free biosensor, in-channel referencing, lab-on-a-chip, organ-on-a-chip

## Abstract

Organ-on-a-Chip systems are emerging as an important in vitro analysis method for drug screening and medical research. For continuous biomolecular monitoring of the cell culture response, label-free detection within the microfluidic system or in the drainage tube is promising. We study photonic crystal slabs integrated with a microfluidic chip as an optical transducer for label-free biomarker detection with a non-contact readout of binding kinetics. This work analyzes the capability of same-channel reference for protein binding measurements by using a spectrometer and 1D spatially resolved data evaluation with a spatial resolution of 1.2 μm. A cross-correlation-based data-analysis procedure is implemented. First, an ethanol–water dilution series is used to obtain the limit of detection (LOD). The median of all row LODs is $\left(2.3\pm 0.4\right)\times 10^{-4}$ RIU with 10 s exposure time per image and $\left(1.3\pm 0.24\right)\times 10^{-4}$ RIU with 30 s exposure time. Next, we used a streptavidin–biotin binding process as a test system for binding kinetics. Time series of optical spectra were recorded while constantly injecting streptavidin in DPBS at concentrations of 1.6 nM, 3.3 nM, 16.6 nM and 33.3 nM into one channel half as well as the whole channel. The results show that localized binding within a microfluidic channel is achieved under laminar flow. Furthermore, binding kinetics are fading out at the microfluidic channel edge due to the velocity profile.

## 1. Introduction

Organ-on-a-Chip (OoC) platforms are an important in vitro analysis tool for parallelized drug development and medical research, for example, on cancer [1] or viral infections [2]. In an OoC platform, a cell culture is realized in a microfluidic system with the aim to achieve a similar response to an entire organ or even an organ system. Different types of cells may be cultivated on OoC platforms, such as lung cells, cardiovascular cells, brain cells, or skin cells [2]. In order to maintain the OoC in the correct environmental conditions and to monitor the response of the OoC to drugs or other stimuli, it is necessary to analyze small amounts of liquid in real-time, with continuous measurements over the course of days or weeks [1]. A successful OoC allows us to predict the organ response to diseases or drugs without affecting the patients’ health [3] or using animal testing [1]. Here, we investigate a biosensor technology that may be suitable for continuous analysis of biomarkers in OoC platforms.

The background of biochip development is reviewed by Azizipour et al. [2]. They describe Lab-on-a-Chip (LoC) devices as the predecessor of OoC devices, being themselves a derivative from micro-total analysis systems (μTAS). All platforms have in common that they use small amounts of sample fluid. LoC devices are used to analyze small amounts of body fluids such as blood, saliva, tears, urine or sweat. In addition, they automate analysis steps and therefore decrease errors in handling. They allow for usage by untrained personnel [4], or even the patients themselves. While both LoC and OoC devices use small sample volumes with integrated biosensors, LoC devices usually aim at the development of autonomous point of care devices, whereas OoC devices are often used in a laboratory context. Therefore, OoC devices typically utilize more sophisticated and costly stationary laboratory equipment.

Microfabrication methods and the development of microfluidic systems enabled the development of on-chip analysis platforms. The sample volumes that fit into and flow through a microfluidic are much lower compared to traditional methods, such as culture dishes [2]. Further advantages are a faster analysis due to fast reaction times [5,6], parallel testing and lower costs [2,7,8]. Because microfluidics have channel sizes in the micrometer range, their Reynolds numbers are usually quite low [2,9,10]. A low Reynolds number indicates the dominance of viscous forces over inertial forces in a fluid stream. Therefore, no mixing in between parallel streams occurs [9,11].

Tølbøl Sørensen et al. [9] used this principle to measure the diffusion coefficients of proteins with a photonic crystal sensor combined with a T-shaped microfluidic. In this label-free measurement method, they recorded the reflected and spatially resolved spectra of the optofluidic system while injecting a sample and a buffer fluid simultaneously with constant pressure. The shift of the resonance wavelength was imaged in a kymograph. For each steady state, they estimated the diffusion profiles by fitting a profile function to the cross-section. Due to the steady-state nature of the measurement, its accuracy can be improved by measuring for longer periods.

Optical sensors are one of many options to analyze the sample fluids [6,10,12]. Several optical sensors are based on fluorescent or chemiluminescent labeling [12]; however, labeling typically involves additional procedures [13] and is therefore more difficult to integrate in a LoC device. In addition, fluorescent proteins can interfere with the cell function [14] or alter the physical properties of the analyzed molecule [9]. The optical detection methods surface plasmon resonance (SPR) and total internal reflection ellipsometry (TIRE) are used in highly sensitive, label-free biosensors, but are high-cost and require bulky equipment [13,15]. Guided mode resonance (GMR) sensors, also known as photonic crystal slab sensors, are easier to miniaturize into label-free biosensors [8,16,17,18,19,20,21]. They can be functionalized [22] with antibodies or aptamers to detect biomarkers such as proteins or small molecules [17,23,24].

Photonic crystal [25] slabs consist of a nanostructured high-index dielectric material on a substrate. The structure acts as a waveguide for the resonant mode. Part of the electric field of the guided mode reaches out of the waveguide and into the superstrate material [15]. This mode fraction is called the evanescent field [26]. The penetration depth of the evanescent field is typically in the order of 0.1–1 μm into the analyte fluid [15]. The sensitive surface of the photonic crystal transduces local changes in the refractive index into a shift in the resonance wavelength. Small molecules such as proteins can be detected when bound to the surface without an extra labeling step. The biofluid can be brought closer to the sensitive surface to avoid long diffusion times by using a microfluidic channel [27]. In addition, as the laminar flow is present in straight microfluidic channels, multiple different fluids can flow side by side. This can be used for referencing or even multiplexing.

Choi et al. developed optical biosensors based on a PCS integrated into a microfluidic to measure protein-binding kinetics in 5 [28] and 11 [29] flow channels simultaneously. They used a spectrometer to measure the spectral response of the channels’ cross-section with a spatial resolution of 22.3 μm and investigated the response of a heparin–lactoferrin assay.

Although binding kinetics [5] and spatially resolved refractive index measurements [9] have been investigated before, the spatially resolved binding kinetics within a microfluidic has not been analyzed in detail. Additionally, the problem of low light conditions while measuring kinetic series with a spectrometer has not been addressed.

In this work, we analyze spatially resolved protein binding kinetics in a microfluidic channel with a photonic crystal biosensor. The channel is sampled continuously with a spectrometer. The shift of the resonance wavelength is estimated with a time delay estimation method [30]. First, the limit of detection of this method is determined by measuring an ethanol–water dilution series. Second, a localized binding experiment is performed using biotin and different concentrations of streptavidin as a test system. Localized binding is obtained by using the laminar flow to inject two parallel streams into the microfluidic channel. This enables referencing within the microfluidic channel. Further, due to the high spatial resolution, we are able to reference in close proximity and to areas of same surface functionalization. This allows for a discriminative analysis of surface effects, which is of interest for material and surface interactions. Finally, binding slopes as well as edge effects due to the microfluidic channel are shown.

This paper is structured as follows. Section 2 contains materials and methods for the device fabrication, Section 3 shows the results as they are being discussed, and conclusions are drawn in Section 4.

## 2. Materials and Methods

**Figure 1:**
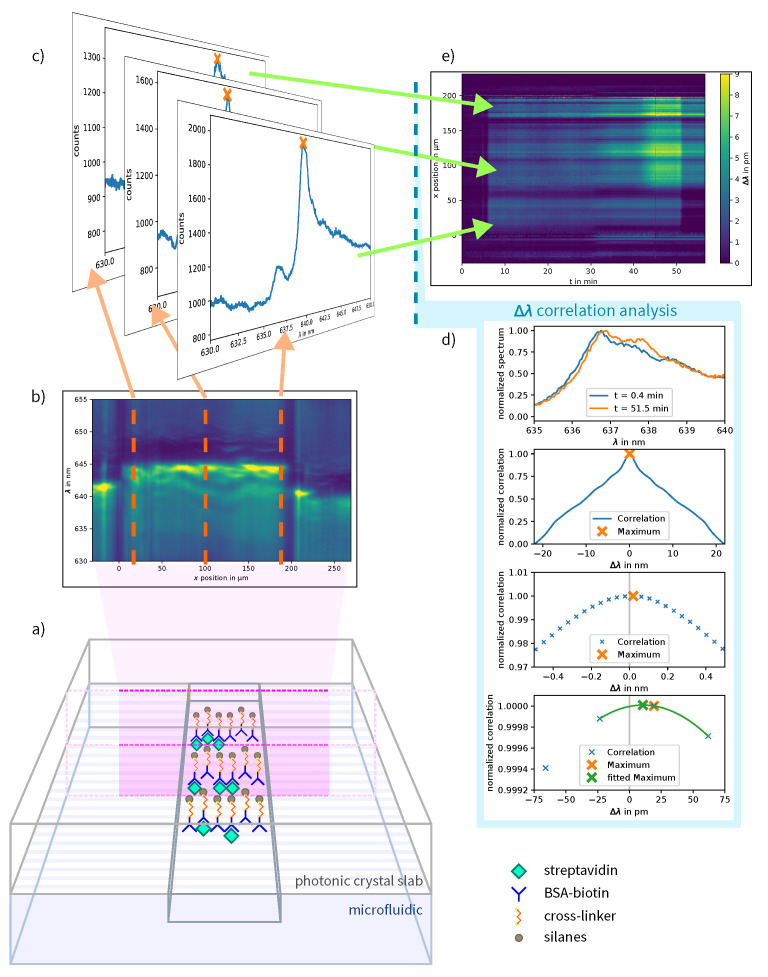
Concept for the measurement and analysis setup. (**a**) Functionalized photonic crystal slab (PCS) with microfluidic channel. (**b**) A cross-section of the microfluidic channel is recorded with a spectrometer and a spatial resolution of 1.2 μm as kinetic series. (**c**) Single spectra at different x-positions. (**d**) The resonance wavelength shift depends on the refractive index change on the PCS’s surface due to protein binding. The shift is calculated for each row by using a cross-correlation based data-analysis procedure. First, the correlation of two spectra recorded at the same position at different times was calculated. The peak of the correlation function is then interpolated with a quadratic function. The position of the peak corresponds to the resonance wavelength shift. (**e**) Kymograph of the biotin-streptavidin binding experiment.

**Figure 2:**
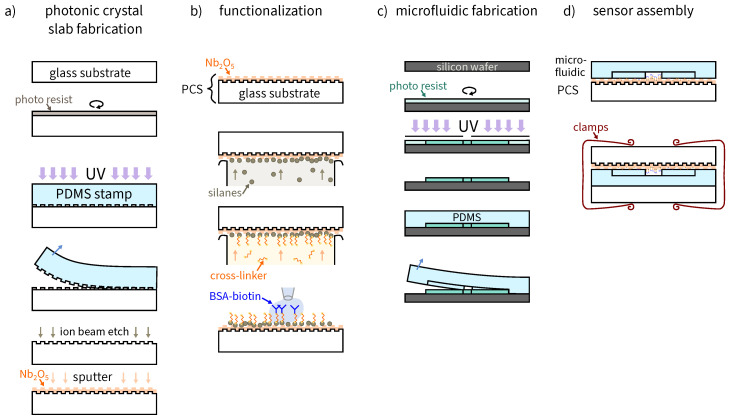
Overview of the fabrication steps for photonic crystal slab (PCS) fabrication, functionalization of the PCS, microfluidic fabrication, and sensor assembly. (**a**) Fabrication of the PCS by spincoating photo resist onto a glass substrate and imprinting the nanostructure with a polydimethylsiloxane (PDMS) stamp. Afterwards, the structure was etched into the glass substrate while removing the photo resist. Niobium pentoxide was sputtered onto the structured substrate. (**b**) The PCS from (**a**) was placed in vacuum, upside down onto a beaker containing (3-Aminopropyl)triethoxysilane (APTES). After subsequent baking, the procedure was repeated with a cross-linking solution. Bovine serum albumin (BSA)-biotin was immobilized onto the surface followed by passivation with BSA in DPBS 1 mg/1 mL. (**c**) The microfluidic was fabricated by structuring SU-8 photo resist on a silicon wafer to create a negative microfluidic master. Following development, the microfluidic was molded from the master with PDMS. (**d**) The sensor was assembled by aligning the microfluidic channel with the BSA-biotin functionalization spot. The sensor was fixed with office clamps.

This section introduces the used materials and explains in brief the fabrication of the photonic crystal and the microfluidic, the functionalization process and the sensor assembly. Finally, the data processing for the analysis is described. The concept for the setup is shown in Figure 1. An overview of the fabrication is shown in Figure 2.

### 2.1. Materials

Polydimethylsiloxane (PDMS) (Sylgard 184, Dow, Torrance, CA, USA) was used to fabricate the microfluidic and the stamp for the UV nanoimprint lithography (UV-NIL) of the photonic crystal. For the nanoimprint, AMOPRIME^®^ (AMO GmbH, Aachen, Germany) and AMONIL^®^ (AMO GmbH, Germany) were used as nanoimprint resist. Niobium pentoxide (Nb${}_{2}$O${}_{5}$) (EJUNBOX353TK4, Kurt J. Lesker, Jefferson Hills, PA, USA) was sputtered onto the photonic crystal [31] as a 100 nm thick high-index layer [32].

SU8-50 (Kayaku Advanced Materials, Westborough, MA, USA) as negative photo resist and PDMS were used to fabricate the microfluidic.

To functionalize the surface of the photonic crystal, silanes (3-Aminopropyl)triethoxysilane (APTES) (440140, Sigma-Aldrich, USA), dry methanol (322415, Sigma-Aldrich, Saint Louis, MO, USA), Dulbecco’s Phosphate Buffered Saline (DPBS) (D8537, Sigma-Aldrich, USA) and a cross-linking solution were used. The cross-linking solution was fabricated with 200 mg PDITC (258555, Sigma-Aldrich, USA) dissolved in 1 mL pyridine (270970, Sigma-Aldrich, USA) and 9 mL N,N-dimethylformamid (DMF) (227056, Sigma-Aldrich, USA) [16]. BSA-biotin (A8549, Sigma-Aldrich, USA) was immobilized onto the photonic crystal and BSA (05470, Sigma-Aldrich, USA) was used for passivation at the end of the functionalization process. For the experiments, ethanol diluted in de-ionized water in concentrations of 5 vol%, 10 vol%, 15 vol% and 20 vol% was used, streptavidin (189730, Sigma-Aldrich, USA) diluted in DPBS in concentrations of 1.6 nM, 3.3 nM, 16.6 nM and 33.3 nM, as well as NaCl 17 mM in de-ionized water.

### 2.2. Photonic Crystal

The manufacturing process for the photonic crystal is described in detail in [31] and follows a standard nanoimprint process with subsequent ion-beam etching. In brief, a PDMS cast was made from a 25 mm × 25 mm glass master to copy the nanostructure. The glass master contained a periodic grating of 370 nm grating period with a duty cycle of 40 % and 45 nm grating depth. The photo resist was spin-coated onto a glass substrate with dimensions 25 mm × 25 mm × 1 mm. The PDMS was then imprinted once into the soft photo resist layer on the substrate, and then the resist was cured with UV light. The photo resist was then removed by ion-beam etching (PC3000, Oxford Instruments, Abingdon, UK) while also transferring the nanostructure into the glass. Finally, a layer of 100 nm thick Nb${}_{2}$O${}_{5}$ was sputtered onto the glass.

### 2.3. Microfluidic Channel

**Figure 3:**
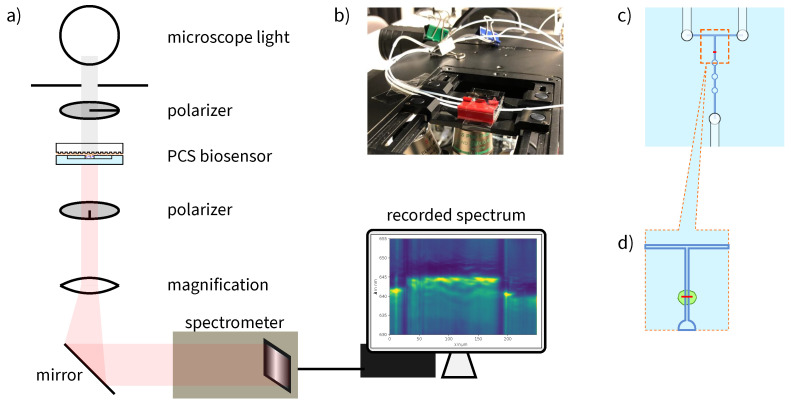
(**a**) Microscope setup of the measurement. The biosensor was placed between polarization filters and the light was measured in transmission with a spectrometer. (**b**) The assembled photonic-crystal biosensor with tubing under the microscope. Office clamps were used to open and close the connected reservoirs from the pressure pump. (**c**) Schema of the microfluidic channel with punching holes for connection of the tubing. (**d**) Close-up of (**c**), measurement position (red) and functionalized area (green).

The microfluidic channel was manufactured by casting PDMS onto a negative microfluidic master. A photo mask was designed for the microfluidic, consisting of a single channel with three larger round chambers with T-shaped access points. The shape of the microfluidic is shown in Figure 3c. The master for the microfluidic was fabricated with a standard SU-8 photo resist process. The wafer was coated with SU8-50 negative resist with a layer thickness of approximately 130 μm, then baked at 65 ${}^{\circ }$C and 95 ${}^{\circ }$C on a hotplate. Then, the photo mask containing the microfluidic’s negative was used to expose the channel regions. After subsequent baking and development steps, PDMS was cast onto the master wafer and cured on a hotplate. The PDMS was peeled from the master wafer and cut into pieces of 25 mm × 25 mm size. The height of the whole PDMS microfluidic was approximately 4–5 mm so that it could enclose the tubing of 1.6 mm diameter from the pressure pump (MCFS™ series, Fluigent, France) during the experiments. The height of the microfluidic channel was measured as approximately 130 μm. The master wafer can be reused to make multiple microfluidics. The microfluidic was used for multiple measurements, since it did not deteriorate over the time of the experiments.

### 2.4. Functionalizing the Photonic Crystal

To use photonic crystals as biosensors, a capture molecule for the biomarker of interest must be immobilized on the surface of the crystal. This is achieved with a functionalization process, which is explained in detail in [16]. In brief, a gas phase evaporation process was used to functionalize the surface. First, a plasma etching step activated the photonic crystal’s surface with oxygen by plasma etching. Then, the photonic crystal was placed with its structure facing down onto the edge of a beaker filled with silanization solution and left in a desiccator in vacuum. The vacuum supports the evaporation of the solution onto the photonic crystal. After 1 h, the photonic crystal was baked for 20 min at 110 ${}^{\circ }$C on a hotplate. The procedure was repeated by placing the photonic crystal on a beaker filled with cross-linking solution in vacuum for 3 h, and then placed under a fume hood for 20 min. Approximately 1 μL of BSA-biotin 1 μg/1 mL solution was immobilized onto the surface using a stencil of the microfluidic for guidance, and then placed into a wet chamber for 1 h. The surface is then passivated by covering it with BSA in DPBS 1 mg/1 mL for 1 h. As a last step, the photonic crystal was rinsed with DPBS and dried with nitrogen.

The sensor can be re-functionalized with the same procedure after cleaning the surface. For cleaning, the sensor is plasma etched with an oxygen flow of 8 sscm at 50 W for 5 min. Afterwards, it is placed in an ultrasonic bath with acetone for 5 min and then isopropanol for 5 min, and dried on a hotplate at 110 ${}^{\circ }$C for 10 min.

### 2.5. Sensor Assembly

The sensor was assembled as follows: the PDMS microfluidic form was pricked out from the top with a 2 mm diameter biopsy punch at the end of the microfluidic channel (outlet) and the ends of the T-shape (2 inlets). Then, a 1.5 mm hole was punched from the side into the 2 mm hole for a sideways connection. Before applying the tubing, the microfluidic was placed on top of the functionalized photonic crystal so that the functionalized area extended over the microfluidic channel’s sidewalls. Another glass substrate was used on top of the microfluidic. Office clamps were applied to the sides of the assembled sensor for fixation.

The tubing for the fluid reservoirs was inserted into the inlet sideways connection of the PDMS and another tube was attached to the microfluidic channel end as an outlet. Then, the biosensor was placed in the microscope setup. The tubing was fixed with tape to prevent moving during the measurement. The pump was set to flush the microfluidic with DPBS buffer at around 4–5 bar to remove air bubbles. Ethanol or isopropanol cannot be used for air bubble removal, as it would damage the functionalization. After the measurement, the sensor can be carefully disassembled without taking damage. It can be re-functionalized for more experiments.

### 2.6. Measurement

The biosensor was placed in a microscope with 20× magnification. The setup is shown in Figure 3. A broad-spectrum lamp (Osram 64623 HLX) was used to expose the photonic crystal.

Crossed polarization filters were used in the path of light in front of and behind the biosensor to suppress background light and obtain a peak in the transmission spectrum instead of a dip [33]. This setup is also used in intensity-based refractive index measurements [16,17]. As a drawback, the intensity of the light is lowered. The light is guided into a spectrometer (Shamrock 500i, Oxford Instruments, UK) by a mirror. A diffraction grating with a grating density of 1199 lines/mm was used. The recorded spectra cover a wavelength range from 619 nm to 662 nm with a resolution of 44 pm.

The spectrometer was setup to record one spectrum per exposure time per position on the slit. Thus, for each of the 256 points on the photonic crystal across the microfluidic channel, a kinetic series was recorded. As protein binding is not a process that can be observed at a steady state, the measurement must be responsive enough to detect the process kinetics with sufficient resolution in time. For the ethanol–water solution measurement, 10 s and 30 s were used as the exposure times and for the streptavidin binding measurement, and 5 s was used as the exposure time.

### 2.7. Data Post-Processing

A shift of the resonance wavelength of the photonic crystal is observed when the biomarkers are bound near the surface. However, changes of the bulk material above the crystal also affect the resonance position. Therefore, a before–after comparison of the resonance position is necessary.

The Python programming language was used to post-process the recorded data. First, outlier values were removed. Then, for the streptavidin binding measurement, each spectrum was added to its subsequent spectrum to have an overall exposure of 10 s. This induces a filtering effect, but improves the LOD of the processed data.

The spectra were analyzed in consecutive order with a cross-correlation algorithm [30]. Better results were obtained using the correlation approach than a maximum estimation of the resonance wavelength by polygon fitting due to the diversity of the acquired spectra across different channel positions. With correlation, each initial shape of the resonance spectrum can be compared to another recorded spectrum during the series. For this, the correlation was calculated between an initial spectrum of each series and each spectrum within the series, for each position across the channel. The position of the maximum of the calculated correlation served as a measure for the shift of the resonance position. The result was plotted as an image, where the resonance shift is color-coded and the positions across the channel are shown over time.

**Figure 4:**
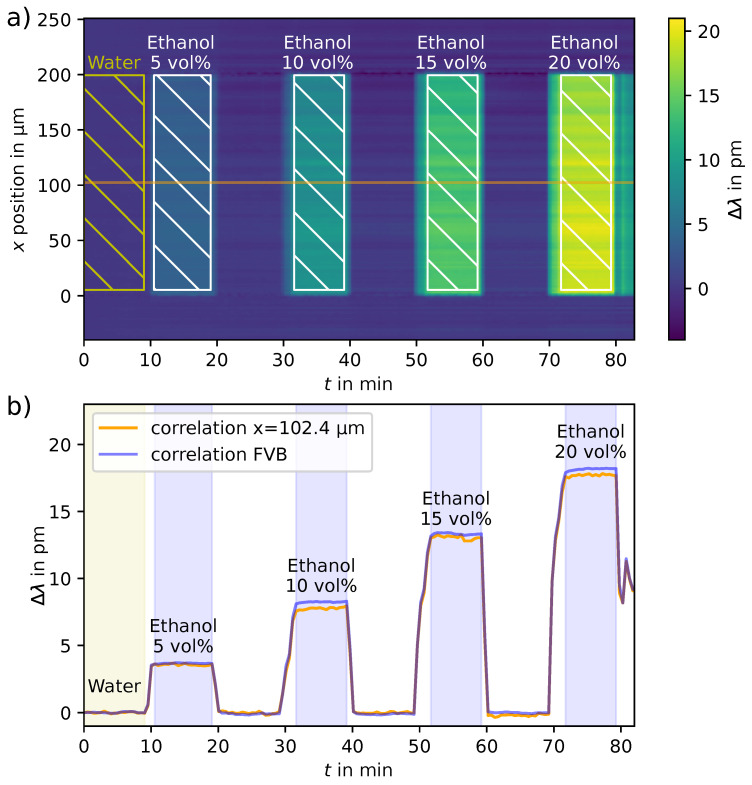
(**a**) Estimated wavelength shift by correlating each spectrum in the series with the first spectrum of the series for each point in time. Here, an image was taken every 30 s. Ranges for the estimation of the limit of detection (LOD) are marked within the image and used for LOD calculation. (**b**) Estimated wavelength shift after full vertical binning (FVB) of the channel region (blue) and the values from a center row for comparison (orange). As the recorded counts per row are relatively low, the processed data are prone to noise and result in a lower limit of detection. By binning all spectra from within the channel area, the LOD improves accordingly. Values from highlighted regions within the microfluidic channel were used for the LOD calculation. The areas above and below these regions are outside the channel region and covered by PDMS.

The resonance wavelength shift can also be plotted as curvature over time. The LOD of a single row is lower compared to a full vertical binning of the spectra due to the lower amount of light that is captured. Nevertheless, because the sample and reference fluids were used in each half of the channel, a binning method for these areas can be used to improve the LOD. For this, all positions referring to a certain area in the channel were summed-up vertically, e.g., all spectra at a given time within a certain position range. This area is marked in the detailed plot in Figure 4. The curves are shown in a second plot next to the image plot.

The LOD of the method was calculated using ethanol steps with vertical binning over the whole channel area. This also means that dividing the channel into multiple parts worsens the limit of detection, as the signal-to-noise ratio (SNR) decreases. The drift was corrected with a linear function before LOD calculation.

It is observed that the refractive index change from NaCl 17 mM in water to DPBS buffer results in different wavelength shifts due to a sensitivity gradient of the photonic crystal. Therefore, this recorded refractive index change was used to normalize the curves from different locations. In addition, the drift was corrected with a linear function. The curves were not normalized for analyzing edge effects in Section 3.2.2, since the curves are also analyzed in PDMS regions where no comparable refractive index change is recorded.

### 2.8. Experiment

#### 2.8.1. Ethanol Concentrations for LOD Calculation

Ethanol–water solutions in different concentrations were used to determine the LOD of the sensor and the analysis method as described above. The sensor was assembled as shown in Figure 2 and Figure 3, and then water and ethanol solutions were injected consecutively. The refractive indices of the solutions were measured by the authors before [16] and the refractive index differences are given in Table 1. The sensor was not functionalized with binding molecules for this experiment. Ethanol in DI water was injected at concentrations of 5 vol%, 10 vol%, 15 vol% and 20 vol% ethanol while alternating injection with pure DI water. Each solution was recorded with 30 s exposure time. The experiment was repeated with 10 s exposure time.

#### 2.8.2. Biotin–Streptavidin Binding

First, the biosensor was flushed with NaCl 17 mM in DI-water with 100 mbar from one inlet while the other inlet was closed with a clamp. Thus, the whole area of the sensor within the microfluidic channel was flushed with NaCl in water. A pressure of 100 mbar was used for all injections in this experiment, which corresponds to a flow rate of approximately 250 μL/min. After 5 min, injection of NaCl in water was stopped and DPBS was injected over the whole area for 5 min. Then, the second inlet was opened and 1.6 nM streptavidin solution injected. Due to laminar flow, only approximately half of the sensor area was exposed to the protein solution. The series was continued with 5 min of DPBS in between streptavidin solutions and the concentration of streptavidin in DPBS increased to 3.3 nM, 16.6 nM and 33.3 nM. The first half of the experiment ended with 5 min of DPBS and 5 min injection of NaCl 17 mM in water. Next, in the second half of the experiment the whole channel was exposed to the same concentration sequence of 5 min of DPBS in between streptavidin solutions and the concentration of streptavidin in DPBS was increased again from 1.6 nM to 3.3 nM, 16.6 nM and 33.3 nM. The second half of the experiment also ended with 5 min of DPBS and 5 min of NaCl 17 mM in water injection.

Since the molecule binding starts to saturate after around 1 min for higher concentration levels, the sample for each imaged spectrum was exposed for 5 s. Thus, a higher time resolution is obtained by the shorter exposure times.

## 3. Results and Discussion

### 3.1. Refractive Index Measurements and LOD Calculation

The limit of detection (LOD) is calculated for each single row within the channel and for every refractive index change. Only steady-state values are used for calculation. The median of all row LODs is $\left(2.3\pm 0.4\right)\times 10^{-4}$ RIU (refractive index units) with 10 s exposure time per image and $\left(1.3\pm 0.24\right)\times 10^{-4}$ RIU with 30 s exposure time. When vertical binning of the spectra within the channel is used, the median LOD is $\left(7.2\pm 1.3\right)\times 10^{-5}$ RIU with 10 s exposure time and $\left(9.4\pm 1.8\right)\times 10^{-5}$ RIU with 30 s exposure time. The correlation analysis of the measurement of ethanol concentrations in water and the areas used for the vertical binning LOD calculation are marked in Figure 4.

It is seen that for the single row, a longer exposure time might result in slightly better LODs. For vertical binning, a three-times improved LOD than for the LOD per row is calculated for the 10 s exposure time, while the improvement is less for 30 s. The measured LODs are in the same order of magnitude and are all sufficient for biomolecular sensing. For the localized analyses in Section 3.2, the spectra are binned as well, and the LOD is assumed to be in between the LOD per row and the LOD for full vertical binning.

### 3.2. Biotin–Streptavidin Binding

**Figure 5:**
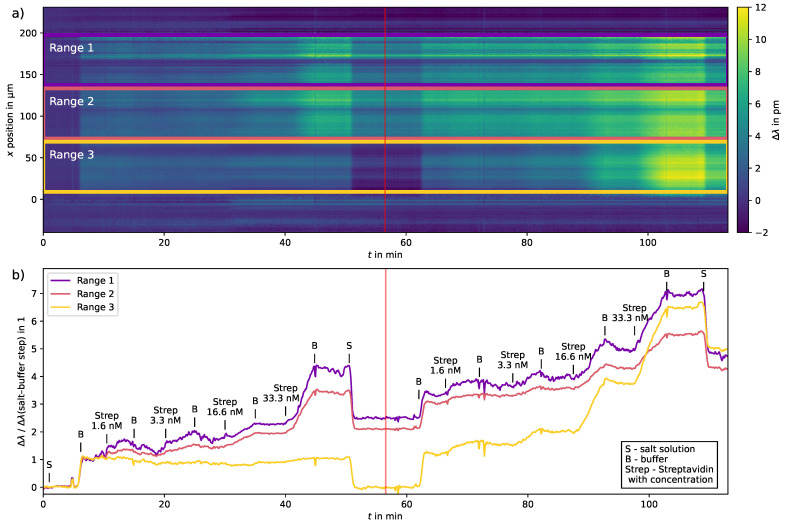
Streptavidin–biotin binding of two consecutive measurements. (**a**) Image plot of the measurement with channel area markings for vertical binning. (**b**) Slopes of vertically binned spectra for the channel areas. Buffer and streptavidin in buffer with increasing concentration were injected simultaneously into the sensor until t=55min. The upper half of the channel shows streptavidin binding whereas the lower part shows little to no binding. The measurement was repeated from t=55min with injecting only one solution at a time to cover the whole channel. The wavelength shift Δλ is normalized to the local wavelength shift from NaCl 17 mM in water to buffer for each range.

The wavelength shift analyses of the streptavidin binding experiment are shown in Figure 5. During the half-sample half-buffer measurement, the sensor moved unnoticed during sample switching. Therefore, the measurement position was slightly shifted and the data were manually corrected afterwards by adjusting the rows. Protein binding was observed in approximately 2/3 of the channel and no binding has been observed in the remaining third. The image shows two single measurements, which have been placed in sequence in order to analyze the binding (measurement 2) when former binding has already occurred (in measurement 1).

Although the applied pressure for simultaneous injection was set to 100 mbar for both fluids, the protein binding occurred in approximately 2/3 of the channel as observed from the results in Figure 5. Therefore, sectioning into thirds was chosen for the vertical binning analysis.

As expected, with the increasing streptavidin concentration, the slope of the curves increases. In the full channel experiment from t=55min, protein binding can also be observed in the previously omitted channel area and shows a strong response to the streptavidin binding.

In the image plot, it can also be observed that stripes of low wavelength shift exist in the channel area. They appear from the first step when the buffer is injected after the injection of NaCl 17 mM in water at t=5min, which indicates that the sensitivity of the photonic crystal is lower at these points. This inhomogeneity is also visible in the ethanol solution measurement in Figure 4, but less prominent. This could be explained by a different sensing spot on the photonic crystal sensor when this binding experiment was performed.

It can be noted that for longer measurements, the drift of the spectra at certain positions increases, which also appears as stripes in the image plot. This can be attributed to the chosen analysis method, because the calculation of the correlation assumes that the form of the spectrum is constant throughout the measurement, which is not the case. Even in areas that were covered with PDMS, this shift can be observed.

The slopes of the binned curves were calculated from sections where streptavidin solutions were injected by linear regression. The slopes are shown in Table 2. They depend on the streptavidin concentration, the association constant and the binding capacity of the ligand [34]. It is seen that the slopes for Range 3 are at first over one order of magnitude lower than for the measurements where binding occurred.

For the consecutive measurement where streptavidin solutions were injected over the whole area, the slopes for the former unbound Range 3 are slightly larger than the bound Ranges 1–2 from the first measurement. We conclude that the sensitivity in this area is larger, also due to less insensitive positions that do not contribute to the wavelength shift. For the second measurement, the slopes for the highest concentration of Ranges 1–2 are lower than in the first run. This indicates a saturation of the binding sites at this point in time.

#### 3.2.1. In-Channel Referencing

**Figure 6:**
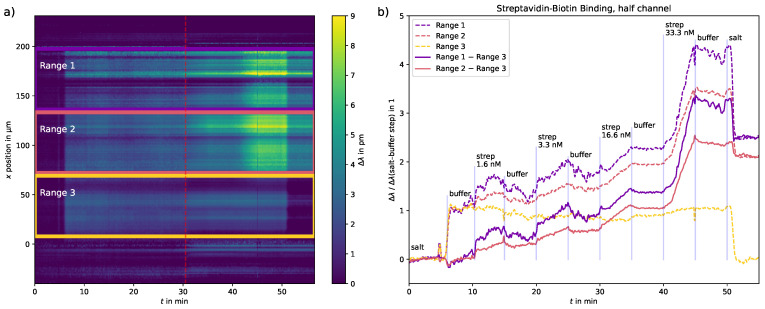
Referencing of the binding sites in Range 1 and Range 2 to the reference site in Range 3. (**a**) First half of the streptavidin–biotin measurement with channel area markings for vertical binning. (**b**) Vertically binned slopes for the channel areas. The dashed lines show the original wavelength shifts of the three ranges, the solid lines show the two binding ranges referenced to the area where only the buffer flowed through and almost no binding was observed. The wavelength shift Δλ is normalized to the local wavelength shift from injection of NaCl 17 mM in water to injection of the buffer for each range.

The spatial resolution of this setup allows in-channel referencing, as shown in Figure 6.

For the first measurement, the two ranges where binding occurred can be referenced to the third region where only the buffer was injected. This way, the binding curves may be corrected, although not perfectly, for possible drifts and systematic errors due to, e.g., switching of the pressure pump. A change in the reference curve indicates possible external influences on the measurement and helps with its interpretation. For example, the signal step from injection of NaCl 17 mM in water to injection of DPBS at t≈51 min indicates for the unreferenced curves, that the wavelength shift is approximately on the same level after the high streptavidin concentrated solution as before. The referenced signals are in part corrected for this large signal step.

#### 3.2.2. Near Channel Edge Protein Binding

**Figure 7:**
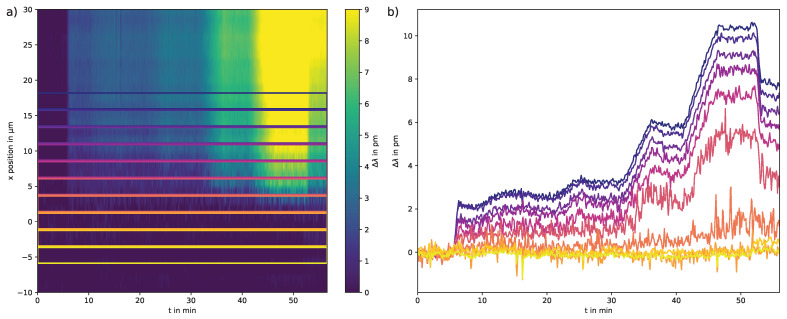
Analysis of the resonance wavelength shift near the channel edge for the full channel measurement. (**a**) Measurement with channel area markings for vertical binning. The section shows part of the former Range 3 close to the channel edge. (**b**) Vertically binned slopes for the channel areas. Ten ranges were selected, overlapping with the edge of the microfluidic channel. The analyzed area covers approximately 1/8 of the whole channel area. Each range contains two binned single row spectra. The overall counts near the edge decrease, therefore the signal-to-noise ratio also decreases, which can be observed for the curves close to the channel edge.

For a rectangular microfluidic channel, a quadratic flow profile is expected [27]. In theory, the flow velocity in the channel decreases from the center to the edge of the channel until it reaches zero. Therefore, near the edge of the channel, the proteins can only diffuse to the binding site, which is slower than the flow velocity. The binding process near the edge was analyzed for the full channel streptavidin injection at the edge of Range 3, where protein binding occurred for the first time. The result is shown in Figure 7.

The binding curves closer to the edge show less prominent binding and more noise as compared to the curves closer to the center of the channel wall. In addition, the spread of these curves indicates a non-linear decrease in flow velocity. We attribute these characteristics to the flow profile.

## 4. Conclusions

In this work, we introduced a microfluidic channel with an integrated photonic crystal slab as a label-free optical transducer for biomolecular binding, a spatially-resolved and temporally-resolved spectral measurement setup and a cross-correlation-based data-analysis approach. With this method we demonstrated that spatially-resolved analysis of the biomolecular binding kinetics in a microfluidic channel is possible.

The slope of the resonance wavelength shift at a particular time indicates the amount of biomarker present. In future work, additional time series analysis is necessary to generate statistical data of association and dissociation for particular biomarkers and their respective capture entities. Biotin–streptavidin has a particularly strong binding affinity with little dissociation [35]. For other biomarkers the kinetics is more complex and may not be mono-exponential [34,36]. As the binding kinetics may be analyzed in detail with the proposed method, the established database may be used to derive an algorithm for calculating the current concentration of a specific biomarker from the binding kinetics with knowledge of the binding history.

Here, the measurement has been demonstrated without temperature stabilization of the sample. This renders the approach of high interest for long-term monitoring of Organ-on-a-Chip platforms. For such an analysis, continuous measurements on the hours to days range are needed that require careful referencing for drift compensation. Here, in-channel drift compensation is possible. Additionally, the approach may be modified in the future to extend the lifetime of the chip by using one region after the other. As shown here, the sensitivity of a specific region may be preserved by flushing it with the buffer until it is needed. If the detection channel is placed in the drainage of the Organ-on-a-Chip platform, it is also feasible to flush it in between for regeneration. It has been demonstrated that a pH change may be used to regenerate aptamer functionalization sites [17].

In conclusion, important progress has been reported on the methodology for the long-term monitoring of Organ-on-a-Chip systems. Combining a label-free detection method with a non-contact optical readout scheme promises minimal impact on the Organ-on-a-Chip system. Next, databases have to be established for the binding kinetics of specific biomarkers to translate the system into application.

## Figures and Tables

**Table 1:** Estimated limit of detection resulting from ethanol concentration measurement.

RIU Step	ΔRIU	Median LOD	Sensitivity	Median LOD	Sensitivity
		10 s	10 s	30 s	30 s
	RIU	RIU	nm/RIU	RIU	nm/RIU
Water-Ethanol 5 vol%	1.98 $\times \;10^{-3}$	6.1 ± 1.1 $\times \;10^{-5}$	1.8	6.0 ± 1.1 $\times \;10^{-5}$	1.8
Water-Ethanol 10 vol%	5.05 $\times \;10^{-3}$	8.0 ± 1.4 $\times \;10^{-5}$	1.6	8.0 ± 1.5 $\times \;10^{-5}$	1.6
Water-Ethanol 15 vol%	7.83 $\times \;10^{-3}$	6.4 ± 1.1 $\times \;10^{-5}$	1.7	10.8 ± 2.0 $\times \;10^{-5}$	1.7
Water-Ethanol 20 vol%	11.42 $\times \;10^{-3}$	9.0 ± 1.6 $\times \;10^{-5}$	1.6	12.9 ± 2.4 $\times \;10^{-5}$	1.6
Median		7.2 ± 1.3 $\times \;10^{-5}$	1.7	9.4 ± 1.8 $\times \;10^{-5}$	1.7

**Table 2:** Slopes during streptavidin injection for different concentrations, half and full channel measurements. The wavelength shift Δλ is normalized to the local wavelength shift from injection of NaCl 17 mM in water to injection of buffer for each range.

Streptavidin	Slopes of Resonance Wavelength Shift					
Concentration	Range 1		Range 2		Range 3	
	Half	Full	Half	Full	Half	Full
in nM	Δλ**/**Δλ(Salt-Buffer Step) in s ${}^{-1}$					
1.66	0.7	1.0	0.6	0.6	−0.2	1.2
3.33	1.5	2.2	0.9	1.4	0.0	2.4
16.6	2.1	5.4	1.7	3.2	0.1	7.1
33.3	9.7	6.3	6.3	3.8	0.4	9.0

## Data Availability

The data are currently not publicly available but may be provided upon reasonable request by the authors.

## Abbreviations

The following abbreviations are used in this manuscript:

DI	de-ionized
BSA	Bovine serum albumin
DPBS	Dulbecco’s phosphate buffered saline
GMR	Guided mode resonance
LoC	Lab-on-a-Chip
LOD	Limit of detection
OoC	Organ-on-a-Chip
PCS	Photonic crystal slab
PDMS	Polydimethylsiloxane
RIU	Refractive index units
UV-NIL	UV nanoimprint lithography
